# Circadian Control of Protein Synthesis

**DOI:** 10.1002/bies.202300158

**Published:** 2024-12-12

**Authors:** Nathan R. James, John S. O'Neill

**Affiliations:** ^1^ Division of Cell Biology MRC Laboratory of Molecular Biology Cambridge UK

**Keywords:** biphasic model, circadian rhythms, protein synthesis, temporal compartmentalization, translational initiation

## Abstract

Daily rhythms in the rate and specificity of protein synthesis occur in most mammalian cells through an interaction between cell‐autonomous circadian regulation and daily cycles of systemic cues. However, the overall protein content of a typical cell changes little over 24 h. For most proteins, translation appears to be coordinated with protein degradation, producing phases of proteomic renewal that maximize energy efficiency while broadly maintaining proteostasis across the solar cycle. We propose that a major function of this temporal compartmentalization—and of circadian rhythmicity in general—is to optimize the energy efficiency of protein synthesis and associated processes such as complex assembly. We further propose that much of this temporal compartmentalization is achieved at the level of translational initiation, such that the translational machinery alternates between distinct translational mechanisms, each using a distinct toolkit of phosphoproteins to preferentially recognize and translate different classes of mRNA.

## Graphical abstract

The circadian clock maintains proteostasis in part through rhythmic control of protein synthesis. We propose that, across the day, translation alternates between distinct mechanisms to optimize energy efficiency and regulate complex assembly. This temporal compartmentalization produces alternating phases of slow specialized translation and rapid proteomic renewal.

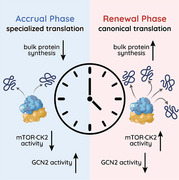

## 1 Introduction

Protein synthesis is the most expensive thing you will ever do. Though there was once a time when primordial life existed without proteins, all modern life is entirely dependent on these foldable strings of amino acids. Proteins are synthesized using a set of biomolecular instructions known as the genetic code. In contrast to replication and transcription, during which the sequence of one nucleic acid is copied to synthesize another, the process of protein synthesis is aptly called *translation* because the language of nucleic acids is translated into the language of protein. The molecular machine responsible for translation is the ribosome—a highly abundant multimegadalton‐sized ribonucleoprotein complex—alongside mRNA, tRNA, and a variety of proteins known as translation factors. By reading the message encoded in an mRNA, the ribosome connects amino acids in a defined sequence to synthesize a nascent protein.

Because proteins are so energetically expensive to produce [1, 2], it is vitally important that translation is controlled in *time*. Much research has focused on the sensitivity of translation to acute stimuli such as growth factors or oxidative stress, but even an isolated cell under constant environmental conditions must regulate the timings of its biomolecular reactions to optimize the cost‐effectiveness of protein synthesis and avoid the proteotoxic consequences of supernumerary subunits and protein aggregation. In many species, this temporal regulation follows the solar cycle of day and night, meaning that the rate of translation oscillates with a period of ~24 h. Even when removed from environmental timing cues, this rhythmic translation persists in both whole organisms and cell cultures [3, 4, 5, 6, 7].

For millions of years, life on Earth has been subjected to a 24‐h cycle of day and night in which environmental factors such as temperature, light intensity, and nutrient availability vary with a predictable rhythm. It is widely believed that a selective advantage is gained from anticipating these variations and adjusting the homeostatic balance of the cell to prepare for times of waking or sleeping, feeding or fasting, and increased or decreased energy expenditure [8]. Therefore, most species have evolved intracellular timekeeping mechanisms known as circadian clocks—from the Latin *circā* (*about*) and *diēs* (*day*)—that keep pace with the solar cycle and anticipate the predictable demands of day and night. Such regulation is especially important in mammals, as most aspects of mammalian physiology vary throughout the day because of interactions between internal circadian clocks and external environmental rhythms [9].

In mammalian cells, the molecular mechanism by which circadian rhythms are generated is commonly thought to involve a negative‐feedback loop known as the transcription–translation feedback loop (TTFL). Emphasis has historically been placed on the *transcription* part of this model and a set of transcription factors known as *clock proteins* [9]. Two of these proteins—BMAL1 and CLOCK—form a heterodimer that activates transcription of several clock‐controlled genes. Some of these genes encode the clock proteins PER1, PER2, PER3, CRY1, and CRY2, which are synthesized in the cytosol and assemble into a series of multimegadalton‐sized complexes [10]. These complexes then enter the nucleus, bind the BMAL1·CLOCK heterodimer, and repress transcription of their own genes. This transcriptional repression is thought to slow the rate at which clock proteins are synthesized. At around the same time, the clock proteins are gradually degraded, causing their intracellular abundances to decrease and eventually relieving the transcriptional repression. A key feature of this model is that inbuilt delay mechanisms—controlled at least in part through phosphorylation by the casein kinases CK1δ, CK1ε, and CK2—prevent the negative‐feedback loop from reaching a steady state. The result is that clock proteins oscillate in abundance with a period of ~24 h, even in single cells under constant environmental conditions [11].

According to the TTFL model, rhythmic transcription of clock‐controlled genes causes rhythmic synthesis of a host of proteins that alter the proteomic landscape of the cell throughout the day. However, despite the popularity of this model, only correlative evidence connects rhythmic transcription to rhythmic changes in the cellular proteome. This has led to a dawning awareness of the importance of translational and post‐translational control in the generation of circadian physiology.

There are several clues that circadian biology is controlled directly at the protein level. In cell cultures, for example, circadian readouts resist large‐scale fluctuations in the rate of transcription [12], yet even comparatively mild inhibition of protein synthesis [13] or protein degradation [14] eliminates detectable rhythms. Furthermore, studies in mice have revealed that the majority of rhythmically abundant proteins are translated from mRNAs with no detectable abundance rhythms [15, 16, 17, 18, 19, 20, 21]. Just as noteworthy are the many proteins that display no rhythms in their abundance or rate of synthesis despite being translated from rhythmically abundant mRNAs [18, 22]. Such observations can only be explained with reference to direct translational control.

This comes as no surprise to those outside the circadian field. In eukaryotes, the correlation between mRNA abundance and protein abundance is notoriously poor [23, 24]. Unlike bacteria, in which transcription and translation are directly coupled, the nuclear envelope of eukaryotes requires that translation be regulated independently of any changes in the nucleus. The canonical model used to explain circadian rhythms suggests that timing information flows through the central dogma—rhythmic transcription of a gene causes rhythmic accumulation of its mRNA, which causes rhythmic synthesis, abundance, and activity of its encoded protein. However, besides a handful of short‐lived and low‐abundance transcription factors, we are aware of no instances where such causality has been directly demonstrated. In the absence of stronger evidence, therefore, we suggest that emphasis should be placed on direct translational control in concert with protein degradation and other post‐translational processes. To be clear, transcription is important—transcription is required for gene expression, after all—but *rhythmic* transcription of clock‐controlled genes appears to be neither sufficient nor necessary for the generation of circadian phenotypes.

This principle extends to the clock proteins themselves. While there is overwhelming evidence that daily variation in clock‐protein activity causes rhythmic transcription of the *PER1* and *PER2* genes, the evidence linking this rhythmic transcription to rhythmic protein abundance is largely correlative. Strikingly, the abundances of PER1 and PER2 proteins continue to oscillate when their mRNAs are transcribed from constitutive nonrhythmic promoters, strongly suggesting an important role for post‐transcriptional and translational control [25, 26].

How, then, does the circadian clock regulate protein synthesis and proteomic composition? This question has been little investigated, but some progress has been made towards a mechanistic answer. In this review, we summarize the current state of knowledge regarding the temporal control of protein synthesis and speculate on some of the mechanisms at work. Building on these ideas, we propose a hypothesis for circadian proteostasis that integrates the contribution of translational control.

## 2 Circadian Control of Proteostasis

### 2.1 Functions of Rhythmic Protein Abundance

It is commonly assumed that observable circadian rhythms are caused by changes in intracellular protein abundance. According to this popular paradigm, rhythms in protein abundance cause rhythms in protein activity, which in turn lead to rhythms in organismal physiology and behavior. However, while this might be true for a small minority of proteins with very low abundances and short half‐lives, such as clock proteins, most proteins are maintained at roughly constant levels in the cell. Where abundance rhythms have been reported to correlate with activity rhythms, the amplitudes of the abundance rhythms are generally too small to account for any changes in protein activity. For example, the abundances of several actin regulators have been shown to oscillate with low relative amplitude (< 10%) despite a twofold variation in actin polymerization and cell motility across the day [7, 27]. Similarly, most metabolic enzymes are expressed at high concentrations such that their abundances are never rate‐limiting [28, 29, 30, 31], yet these same enzymes are responsible for well‐characterized circadian rhythms in metabolic reactions [32]. Rather than protein abundance being the deciding factor, these circadian phenotypes are instead generated at the post‐translational level by processes such as protein phosphorylation, allostery, and substrate localization [33].

The problem of *effect size* is further exacerbated by the intrinsic variability of supposedly identical cells. Protein abundances reportedly vary by up to 10% between cells in the same culture at the same time [34, 35, 36, 37, 38]. By comparison, cell‐autonomous circadian rhythms in protein abundance tend to be very modest, showing a median variation of ∼5% across all detected proteins, rising to ∼20% for the ∼7% of detected proteins with statistically significant abundance rhythms [39]. It is difficult to see how any abundance rhythm with an amplitude approximating the intrinsic variation within a population of cells would have functional consequences. The story is somewhat different in vivo, where the influence of daily systemic cues leads to higher‐amplitude rhythms in protein abundance (with a median amplitude of ∼40% for the ∼6% of proteins displaying rhythmic abundance in murine liver), though it should be noted that the identities of “clock‐controlled” proteins and genes are poorly reproducible between laboratories [40, 41].

If we look beyond the circadian field, we find many more examples of poor or no obligatory correlation between protein abundance and protein activity. Take the cell cycle, for example, which shares many features with the circadian cycle and similarly involves time‐sensitive proteomic oscillations [42]. The cell cycle is driven forwards by an engine composed of cyclins and cyclin‐dependent protein kinases. The cyclins are so called because they cycle in abundance; cyclin B, for example, rises from the start of S phase, peaks during mitosis, and is rapidly degraded before cytokinesis. In partnership with CDK1, cyclin B drives entry into M phase and its oscillating abundance is thought to prevent premature mitosis and enable the daughter cells to resume growth after cell division. According to the textbooks, this oscillation is necessary for progression through the successive phases of the cell cycle. However, this oft‐cited model was refuted by some of the earliest experiments on cyclins. In the fission yeast *Schizosaccharomyces*, temperature‐sensitive‐mutants of Cdc13 (the orthologue of cyclin B) or Cdc2 (the orthologue of CDK1) fail to enter M phase when grown at a restrictive temperature, instead appearing as grossly elongated cells stuck in G_2_ phase [43]. Multicopy *overexpression* of *cdc13* or *cdc2*, however, results in no mutant phenotype [44, 45]. Rather than causing premature entry into M phase or mitotic arrest, as might be expected if protein activity were proportional to protein abundance, the cells are instead wild type in appearance. This is because the rhythmicity of Cdc2 activity is conferred not by Cdc13 but by a cell‐autonomous rhythm in protein phosphorylation [46]. Though Cdc13 is also necessary for Cdc2 activation, the abundances of both proteins are largely irrelevant [44‐45, 47]. Neither is degradation of Cdc13 necessary for cell‐cycle progression; constitutively expressing the cyclin by deleting subunits of the anaphase‐promoting complex [48] or by replacing all endogenous cyclins and Cdc2 with a degradation‐resistant Cdc13·Cdc2 fusion protein [49] allows the cell cycle to persist with an almost wild‐type appearance. For the cell cycle, therefore, rhythms in protein abundance are not required for rhythms in protein activity—so long as enough copies of a protein are present, rhythmic phenotypes are generated entirely at the post‐translational level.

What, then, is the function of protein abundance rhythms? Cyclins clearly do cycle, even though this cycling is not required for cell‐cycle progression. Part of the answer seems to be *robustness*, or tolerance of cellular processes to perturbations or fluctuations in protein activities. Cdc13 is not sufficient for Cdc2 activation, but it is *necessary*, so degrading Cdc13 at the same time as inhibiting Cdc2 serves to buffer the cell cycle against errors in the temporal control of Cdc2 activity. Rather than generating a rhythm, Cdc13 amplifies the post‐translationally generated rhythm to protect it against noise.

The same argument can be applied to the circadian cycle and clock proteins such as PER2. Much like cyclins, these proteins cycle in abundance, yet their abundance rhythms are not essential for their circadian functions. Thus, it has recently been shown that constitutive nonrhythmic expression of degradation‐resistant PER2 is sufficient to restore circadian rhythms in locomotor activity and gene expression to *PER1* ^−/−^ *PER2* ^−/−^ *PER3* ^−/−^ triple‐knockout mice [50].

Other clock proteins, such as CRY1 and CRY2, appear to be dispensable for rhythm generation, with protein abundance rhythms persisting in *CRY1^−/−^CRY2^−/−^* double‐knockout cells [39, 51]. Intriguingly, these cells also reveal another potential function of cycling proteins—*amplitude damping*. In the absence of CRY1 and CRY2, in cell culture and in vivo, the abundance rhythms of many proteins increase in amplitude and the overall composition of the proteome is substantially different when compared to wild‐type cells [17, 39]. Comparable observations have been made at the transcriptional level in the flowering plant *Arabidopsis*: overexpression of the clock protein CCA1 disrupts the TTFL yet causes thousands of mRNAs to start cycling in abundance under exogenous light–dark cycles [52]. Though these observations seem counterintuitive at first, they make sense if the primary function of cycling proteins is to maintain cellular homeostasis despite major variations in the rates of transcription, translation, and degradation of mRNA and protein [40]. In other words, the oscillation of a few cycling proteins might serve to offset large‐scale oscillations in cellular metabolism, thereby protecting the cell from proteotoxic stress. Exactly how this damping is achieved remains to be seen, but it presumably involves temporal coordination between protein synthesis and protein degradation [7]. The functions of these antagonistic processes are explored in the next section.

### 2.2 Functions of Rhythmic Protein Synthesis

Whether we consider the circadian cycle or the cell cycle, only a small minority of proteins have been demonstrated to show functionally significant abundance rhythms. Though the mechanism has yet to be characterized in detail, one important function of this select group of cycling proteins may be to dampen the abundance rhythms of other proteins, thereby maintaining steady‐state proteostasis. This is necessary because, unlike bulk protein abundance, the overall rate of protein synthesis varies as much as twofold in vivo and cultured cells [5‐7, 53‐54]. When decoupled from rhythmic protein degradation, as in *CRY1^−/−^CRY2^−/−^* knockout cells, these underlying rhythms in protein synthesis apparently lead to increased oscillations in protein abundance that perturb normal cellular proteostasis [39].

This observation begs a question: If proteostasis requires steady‐state abundances for most proteins, why is protein synthesis rhythmic at all? A clue to an answer comes from the budding yeast *Saccharomyces*. In these cells, most cellular processes undergo ultradian rhythms which cycle more quickly than circadian rhythms and are not synchronized with the solar day. Remarkably, under nutrient‐limited conditions, *Saccharomyces* cultures spontaneously synchronize their individual rhythms to produce robust population‐wide rhythms in oxygen consumption and metabolism known as yeast respiratory oscillations (YROs) [24, 55]. For most of its cycle, a YRO is characterized by low oxygen consumption and increased autophagy, as glucose and amino acids are sequestered and stored. Following this phase, once their intracellular stores are replete, the cells simultaneously enter a phase of high oxygen consumption driven by rapid protein synthesis and aerobic respiration [55].

*Saccharomyces* cells spend most of their energy budget on active transport and protein synthesis, meaning that growth is highly sensitive to nutrient availability. To balance these opposing demands, the cells compress their metabolically intensive processes into periodic bursts of bioenergetic activity. This pulsatile metabolism also occurs in the circadian rhythms of mammals and other eukaryotes, where bulk protein synthesis rises and falls in synchrony with carbohydrate catabolism, aerobic respiration, and organismal activity [22]. We propose, therefore, that the primary ancestral function of both YROs and circadian rhythms is the *temporal compartmentalization* of metabolic processes, and especially protein synthesis, to optimize energy efficiency.

Temporal compartmentalization has two main advantages. The first and most important relates to the rhythmic control of *proteomic renewal*. In quiescent mammalian cells, the cell‐autonomous circadian rhythm of bulk protein synthesis seems to be coordinated with a concomitant rhythm of bulk protein degradation [7]. Consequently, protein turnover occurs in bursts, with old proteins being cleared at the same time as their replacements are synthesized. This ensures that amino acids are available for protein synthesis while minimizing the potential for proteotoxic fluctuations in protein abundance. Such synchronized control would be especially important for proteins with half‐lives greater than 24 h—the majority of mammalian proteins [56]—which gradually and stochastically accrue damage yet must be maintained at steady‐state levels. There are also reports that protein damage might occur rhythmically, following oscillations in the build‐up of reactive oxygen species [57], further suggesting that synthesis and degradation might be synchronized with the phase of greatest oxidative stress.

The second advantage relates to the assembly of *heteromeric macromolecular complexes*. All proteins make specific interactions with other proteins and macromolecules such as RNA and most are found in at least one stable complex. Newly synthesized proteins must be correctly localized to subcellular compartments and assembled into these heteromeric complexes in their correct stoichiometries, or else are wastefully degraded to avoid deleterious aggregation. In the case of highly abundant complexes such as ribosomes, the assembly process involves many tightly controlled steps that are separated from each other by space and time [58]. This spatiotemporal separation allows the proteins to fold together, step by step, into their native structures. Congruent with this, many protein subunits display rhythmic synthesis in phase with their subunit partners; examples include ribosomes, mitochondrial membrane complexes, and even the photosynthetic machineries of plants [59]. The importance of controlling complex assembly is further illustrated by the array of quality‐control mechanisms that have evolved to selectively degrade mislocalized proteins and supernumerary orphan subunits [60]. Mutations that disrupt targeting sequences or alter the balance of protein subunits are known to overburden these quality‐control mechanisms and cause proteotoxic diseases such as thalassemia and neurodegeneration. Notably, *CRY1^−/−^CRY2^−/−^* double‐knockout cells display signs of proteotoxic stress, and the live mice, though viable, suffer comorbidities ranging from growth defects and chronic inflammation to cancer, implying that the *mistiming* of protein synthesis similarly overburdens these quality‐control pathways [39].

A third and more speculative advantage relates to the concept of *hormesis*, which describes a dose‐response relationship where low doses of toxins or stressors improve survival when organisms are later exposed to higher doses [61]. This results in a characteristic inverted‐U‐shaped profile in which cells exposed to zero dose or high dose are more sensitive to external stressors while low‐dose exposures are beneficial. In the case of ultradian and circadian rhythms, cycling through phases of low and high metabolic activity would precondition the cell—and particularly its proteostatic machinery [62]—to remain responsive to sudden environmental changes in temperature, hormonal signalling, nutrient availability, and bioenergetic demand. In other words, there may be a selective advantage conferred on cells that can anticipate and accommodate a changing internal environment because it improves their chances of surviving both predictable and unpredictable changes in the external world. Note that, for both YROs and circadian rhythms, the processes that are routinely upregulated during the phase of low metabolic activity include typical stress responses such as autophagy and macromolecular condensation [6, 55, 63‐66], which might precondition cells to respond faster and more effectively to acute stresses.

Rhythmic protein synthesis appears to be a conserved feature of eukaryotic cells due to the presumed selective benefits of temporal compartmentalization. However, the mechanisms underlying this rhythm remain elusive. For the remainder of this review, we explore the circadian control of translation and speculate on the mechanisms at work.

## 3 Circadian Control of Translational Initiation

### 3.1 A General Outline of Translational Initiation

Translation is a cyclic process that can be conceptually divided into four phases: *initiation*, *elongation*, *termination*, and *recycling*. Though regulation occurs in all phases, control is primarily exerted at the level of initiation before a nascent protein is synthesized. To understand circadian translational control, we must therefore begin with an outline of translational initiation in mammalian cells.

Ribosomes are rarely, if ever, naked. Upon emerging from the recycling phase, the *ribosomal small subunit* (also called the *40S subunit*) is coated with a handful of recycling factors that also participate in translational initiation [67]. The most notable of these is eIF3j, which helps to displace the old mRNA but is also a substoichiometric component of the multifunctional initiation factor eIF3. This thirteen‐subunit protein complex has a star‐shaped core with long extensions that wrap around the ribosome like a starfish engulfing its prey. This starfish is joined by other initiation factors, namely eIF1, eIF1AX, the eIF2 heterotrimer, and eIF5, alongside the methionylated initiator tRNA (Met‐tRNA_i_^AUG^), to form a stable intermediate known as the *43S preinitiation complex* [68]. With slight modifications, this complex is the central machine that drives most mechanisms of translational initiation.

**FIGURE 1:**
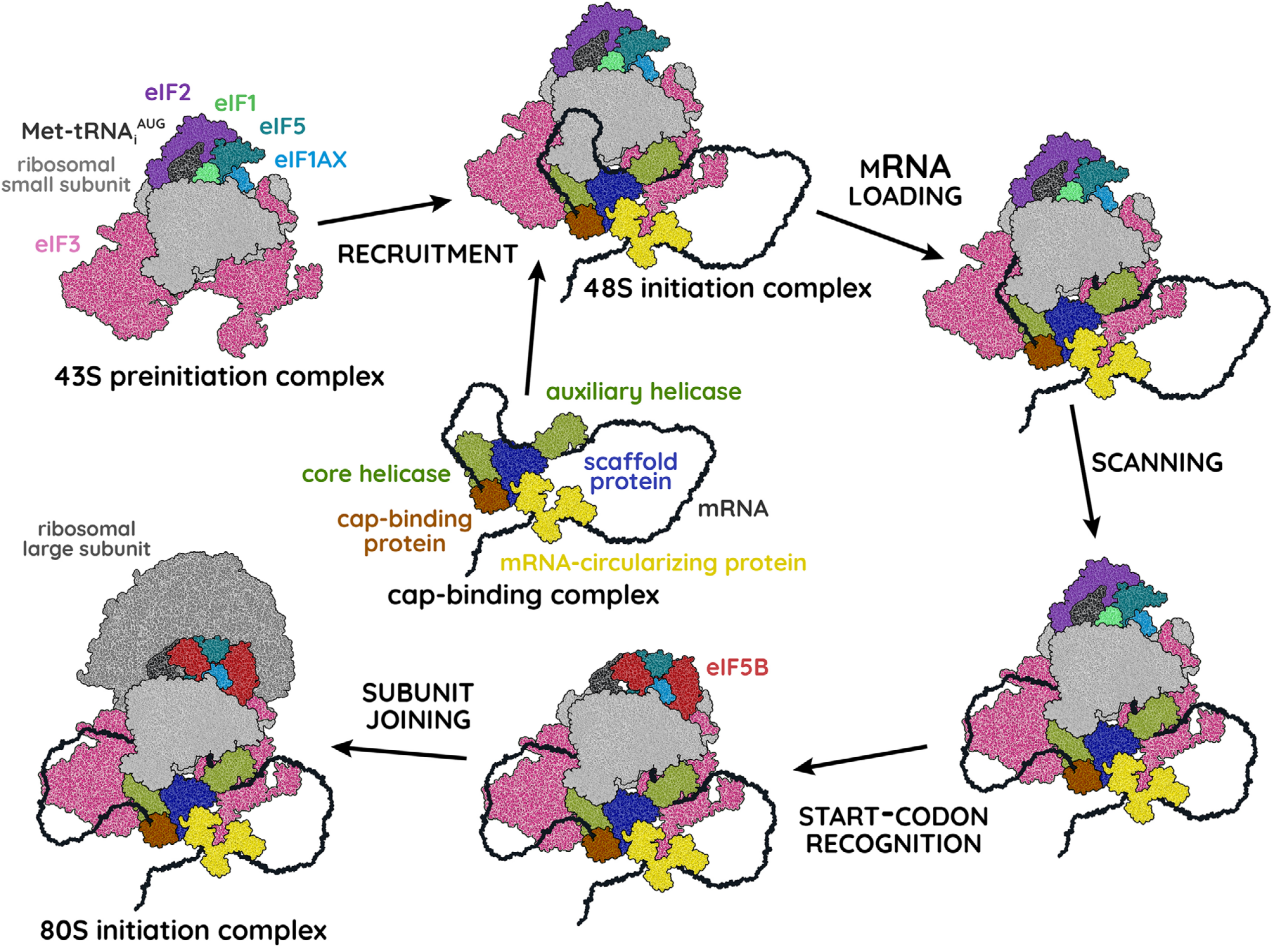
A generalized mechanism of translational initiation, highlighting the major events and core machinery. The cartoon outlines of protein and RNA are based on resolvable regions of published 48S and 80S initiation complexes [84, 242], supplemented with AlphaFold predictions of any missing proteins [243, 244]. Not all initiation mechanisms follow this scheme exactly. In particular, not all mechanisms include the scanning step or use eIF2 to recruit the Met‐tRNA_i_^AUG^. The timing of release of the cap‐binding complex might also vary but seems to occur later during translational elongation in at least some circumstances [245].

Having assembled the 43S preinitiation complex, the first step of initiation proper is recruitment of this complex to the target mRNA, forming the *48S initiation complex* (Figure 1). This is the step that varies most between different initiation mechanisms, but we can describe some general principles here. Each mRNA is usually associated with a *cap‐binding complex*, also known as eIF4F, which assembles around the *N* ^7^‐methylguanosine (m^7^G) cap at the 5′ end of the mRNA [69]. Cap‐binding complexes typically contain a large scaffold protein, a cap‐binding protein, an mRNA‐circularizing protein, and two RNA helicases, in addition to other regulatory factors (Table 1). The role of scaffold protein, forming the structural core of the cap‐binding complex, is usually taken by a member of the eIF4G family, though other more exotic proteins such as the Thr‐tRNA synthetase ThrRS1 can also take on this role [70]. Even more candidates exist for the cap‐binding protein, including eIF3d [71], PARN [72], NCBP2 [73], NCBP3 [74], and the four members of the eIF4E family [75, 76], all of which bind m^7^G, while proteins such as YTHDF3 might fulfil a similar role by instead binding *N* ^6^‐methyladenosine (m^6^A) residues within the mRNA [77, 78]. Occasionally, translation can be cap independent, bypassing the need for a cap‐binding protein altogether, but cap‐independent recruitment is far less common than a cursory glance at the literature would suggest [79]. Together, the scaffold protein and the cap‐binding protein recruit the 43S preinitiation complex to the mRNA.

**TABLE 1:** Representative cap‐binding complexes specific to different mechanisms of translational initiation in mammalian cells.

Mechanism	Cap‐Binding Protein	Scaffold Protein	mRNA‐Circularizing Protein	Core Helicase	Auxiliary Helicase	Additional Factors
canonical initiation	eIF4E1a	eIF4G1	PABP1	eIF4A1	eIF4A1, DHX29, DHX33, etc.	eIF4B or eIF4H PAIP1 MNK1 or MNK2 BMAL1
eIF3d‐mediated initiation	eIF3d	eIF4G2	?	?	?	?
leaky scanning	—	eIF4G2	?	eIF4A1	eIF4A1, DDX3X, DHX9, DHX36, etc.	?
reinitiation	—	eIF4G2	?	eIF4A1	DDX3X	?
RISC‐mediated initiation	PARN	eIF4G2	AGO2	?	?	FXR1
m^6^A‐mediated initiation	YTHDF3	eIF4G2	?	eIF4A3	?	YTHDF1 YBX1 ABCF1
hypoxic initiation	eIF4E2	eIF4G3	LARK1	eIF4A1	?	HIF2α
MNK‐deficient initiation	eIF4E3	eIF4G1	PABP1	eIF4A1	?	?
pioneer initiation	NCBP2	eIF4G1 or eIF4G3 or CTIF	PABP1	eIF4A3	DHX9	NCBP1 SKAR DDX19B
ThrRS1‐mediated initiation	eIF4E2	ThrRS1	PABP1	eIF4A1	?	?

*Note*: Many of these complexes are still poorly characterized. Note that the noncanonical scaffold protein eIF4G2 never interacts with eIF4E1a, PABP1, or their relatives.

The recruitment step is also thought to involve communication between the 5′ and 3′ ends of the mRNA in what is known as the *closed‐loop model* of translational initiation [80]. This communication is mediated by an mRNA‐circularizing protein, which brings the 3′ end of the mRNA into proximity with the 5′ end to further stimulate translation. Members of the PABP family often fulfil this role, but other RNA‐binding proteins such as LARK1 [81] and AGO2 [82] reportedly also mediate circularization. Indeed, any protein that binds the 3′ untranslated region (UTR) of an mRNA could form a closed loop and stimulate translation, at least in principle [83].

In the following step, *mRNA loading*, the mRNA loops over the head of the ribosomal small subunit and slots into the mRNA channel [84]. This creates a blind spot near the 5′ end of the mRNA where potential start codons are ignored. Covering about forty residues in canonical initiation, the length of this blind spot depends on the spatial arrangement of initiation factors and probably varies between different mechanisms of translational initiation. The process of mRNA loading is coordinated by eIF3 in concert with RNA helicases. Though the ribosome is thought to have intrinsic helicase activity, able to unravel some mRNA structures, translation is nonetheless heavily dependent on *trans*‐acting RNA helicases. Recent data have revealed that two such helicases jointly participate in translational initiation. The first of these, which we term the *core helicase*, seems invariably to belong to the eIF4A family of DEAD‐box RNA helicases (Table 1). This helicase has a structural role, connecting the scaffold protein to eIF3 on the distal side of the ribosome [84], and might also resolve weak mRNA structures near the 5′ end. The second RNA helicase, which we term the *auxiliary helicase*, binds the ribosome directly on its proximal side, near the entrance to the mRNA channel, where it presumably helps with mRNA loading. Though only the canonical eIF4A1 has been directly observed at this position in a 48S initiation complex [85], other RNA helicases are known to bind the ribosome at this site and participate in translational initiation, especially when the 5′ UTR is highly structured [86]. Notably, the existence of many DEAD‐box RNA helicases in bacteria, including orthologues of eIF4A1 [87], suggests that the role of auxiliary helicase might be more ancestral, with the core helicase appearing later in eukaryotes only after the evolution of eIF3 and the cap‐binding complex.

The remaining steps—*scanning*, *start‐codon recognition*, and *subunit joining*—are better conserved between initiation mechanisms. From its initial position, the 48S initiation complex moves along the mRNA in the 5′‐to‐3′ direction, scanning for a start codon as it goes [68]. The process of scanning is subject to its own special kinds of regulation which we discuss in Section 3.4. Upon successful recognition of a start codon, the head and body of the ribosomal small subunit close around the mRNA and the complex remodels itself as it commits to translational elongation. Lastly, eIF1AX and eIF5B recruit the *ribosomal large subunit* (also called the *60S subunit*) to form the mature *80S initiation complex* and ready the ribosome for protein synthesis.

As should be apparent, the choice of which mRNA to translate—and how many times to translate it—depends primarily on the recruitment step of translational initiation. Thus, while other steps are conserved both in mechanism and machinery, the recruitment step diverges into several mechanisms and a veritable menagerie of alternative cap‐binding complexes, each of which associates preferentially (though not necessarily exclusively) with a specific class of mRNAs defined by conserved sequence motifs [69, 88]. Within each class, the translational activity of an mRNA depends partly on its relative abundance and partly on its affinity for initiation factors. The translational activity of a whole class, however, is determined by the ability of its preferred cap‐binding complex to recruit ribosomes, which varies with composition and post‐translational modification. Though evidence is currently limited, we propose that this is how the circadian clock controls translation and proteostasis: by rhythmically modifying cap‐binding complexes to alternate between competing mechanisms of translational initiation, thereby favouring translation of different classes of mRNA at different times. In the following sections, we discuss the evidence for circadian control of some of these mechanisms.

### 3.2 Canonical Initiation

What is generally referred to as the *canonical* mechanism of translational initiation is perhaps better called the *mTOR‐directed* mechanism because it is primarily activated by mTOR signalling. Originally identified as the target of rapamycin (TOR) in the budding yeast *Saccharomyces*, the twin proteins Tor1 and Tor2 and their single mammalian orthologue mTOR are highly conserved protein kinases and signalling hubs that control growth and cell division in response to—or in anticipation of—nutrient availability, energy status, and extracellular signals [89]. Just some of the major regulators of mTOR signalling include GCN2 and the Ragulator complex (sensing amino‐acid availability), AMPK (sensing ATP availability), receptor tyrosine kinases (sensing extracellular growth factors and mitogens), GSK3β (responding to WNT signalling), p53 (sensing DNA damage), and DDIT4 (sensing hypoxia).

In mammals, mTOR activity is particularly sensitive to growth‐factor stimulation. The transition from the fasted to the fed state leads to a systemic increase in growth‐factor signalling that potently activates mTOR across a range of mammalian cell types and tissues [90]. In nocturnal animals such as mice, most feeding and locomotor activity occurs at night. As a result, in the tissues of mice kept in cycling photic conditions (that is, cycles of 12 h light and 12 h dark), mTOR activity is elevated during the dark phase [91]. Under constant darkness, however, centrally organized rhythms in activity and feeding continue to anticipate the timing of the dark phase, with the consequence that daily rhythms in mTOR signalling persist in the absence of any environmental timing cues. Indeed, the cell‐autonomous nature of circadian rhythms means that approximately daily rhythms in mTOR activity persist in cultured cells and tissues under constant conditions and in the absence of periodic growth‐factor stimulation [6, 92, 93]. The precise mechanism by which cell‐intrinsic mTOR rhythms arise remains unclear, though evidence exists for direct rhythmic regulation by cytosolic magnesium [53], PER2 [94], and AKT1 [95], any or all of which might be relevant.

The mTOR protein itself exists in two similar but functionally distinct complexes: mTORC1 and mTORC2. The latter complex is less well characterized, but mTORC2 reportedly interacts with elongating ribosomes and phosphorylates nascent proteins as they emerge from the exit tunnel [96]. Binding to ribosomes—even when translationally silent—also increases the kinase activity of mTORC2 towards mature proteins such as AKT1, leading to the hypothesis that mTORC2 senses the number of ribosomes in the cell and adjusts the rate of protein synthesis accordingly [97]. By contrast, the well‐characterized mTORC1 is generally considered to be the central signalling hub of the mTOR pathway. Activation of mTORC1 is thought to require its association with the lysosomal membrane, where it integrates various signals of cellular state and regulates many biological processes via a handful of potent downstream effectors.

In vivo and ex vivo, daily rhythms in mTOR activity strongly correlate with, and are thought to cause, rhythms in bulk protein synthesis that can vary as much as twofold across the day. Of the vast number of proteins responsive to mTOR signalling, many are regulators of ribosome biogenesis and bulk translation. Both processes show circadian regulation in murine liver and cultured cells in phase with rhythmic mTOR activity [6, 98]. Some mTOR‐sensitive regulators include the transcription factors TIF‐IA [99], MAF1 [100, 101], and ATF4 [102], the translational elongation factors eEF1A1 [103] and eEF2 [104], and the RNA‐binding proteins NCBP1 [105] and SKAR [106] that participate in the pioneer round of translation on newly synthesized mRNA. By controlling the activities of such proteins, mTOR increases both the biogenesis of ribosomes and the overall rate of translation [98, 107]. Conversely, suppression of mTOR activity leads to a cell‐wide slow‐down of protein synthesis [108] and an increased rate of autophagy, including ribophagy [109].

**FIGURE 2:**
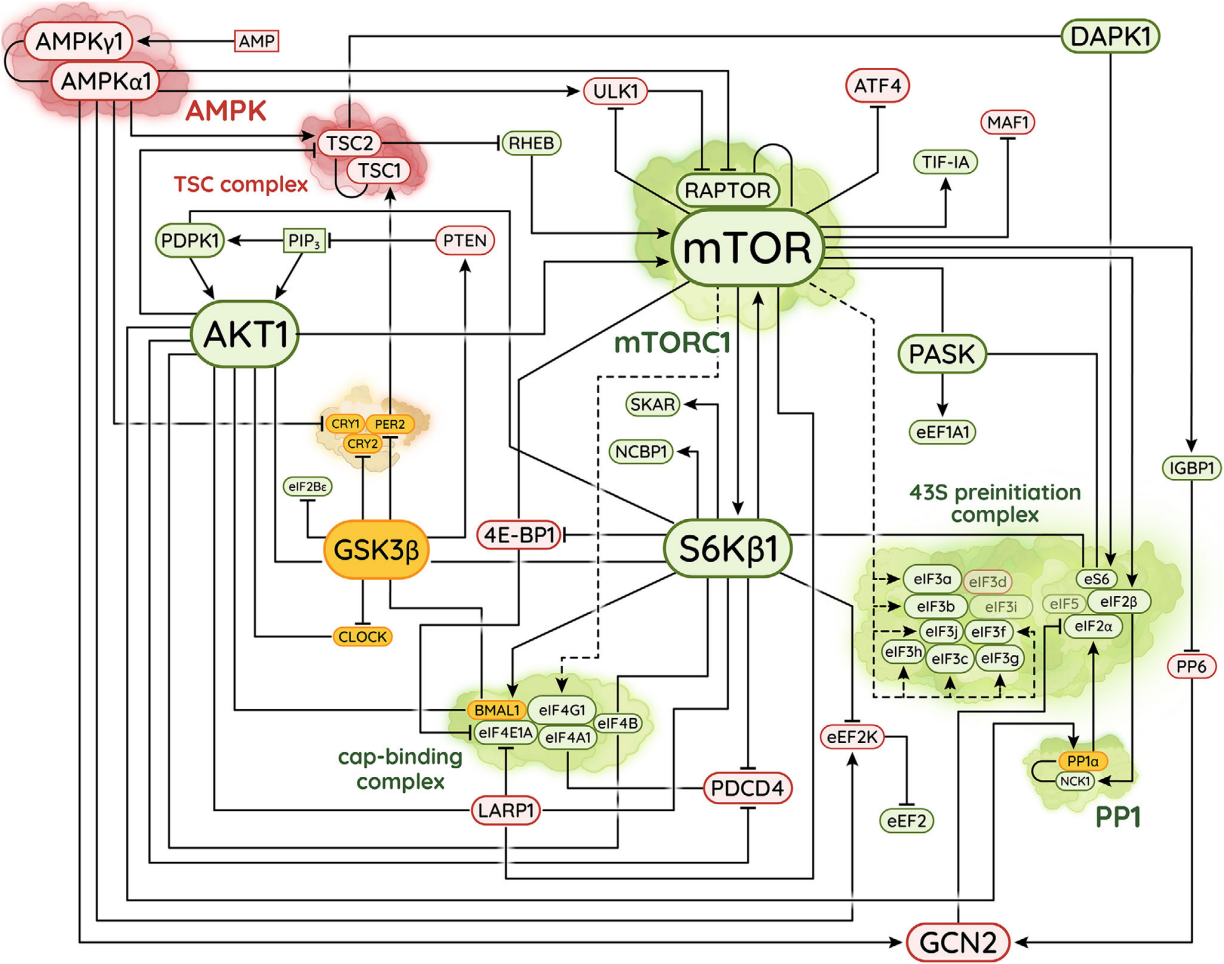
The mTOR signalling pathway, highlighting mTORC1 and interactions that regulate canonical initiation. **Green** shows procanonical factors, **red** shows anticanonical factors, and **orange** shows proteins implicated in the circadian TTFL (which may promote or suppress canonical initiation, depending on context). Solid lines represent direct interactions; dotted lines represent interactions that might be indirect. For regulatory proteins with multiple paralogues, such as AKT1, S6Kβ1, and 4E‐BP1, the related proteins are assumed to function similarly and only one paralogue is shown. Note how multiple branches converge on the cap‐binding complex and the 43S preinitiation complex, which must come together for translational initiation.

Though this large‐scale control of *translational activity* must contribute to circadian proteostasis, the most important effects of mTOR are mediated through *translational specificity*. Plotting the mTOR pathway as a flowchart (Figure 2) reveals that many of its downstream targets converge on the canonical cap‐binding complex and the 43S preinitiation complex, which together drive translational initiation. By controlling the activities and compositions of both complexes in parallel, mTOR selectively upregulates canonical initiation while suppressing alternative mechanisms.

Canonical initiation closely follows the general outline of translational initiation described earlier but differs in some details and its mode of regulation. The mechanism is most clearly distinguished from others by its use of the canonical cap‐binding complex, minimally comprising eIF4A1, eIF4E1a, and eIF4G1 (Table 1). These proteins also associate with eIF4B or its smaller relative eIF4H, both of which are poorly understood but seem to enhance the helicase activity of eIF4A1. Alternatively, instead of recruiting eIF4B or eIF4H, the second copy of eIF4A1 is sometimes replaced by a more potent auxiliary helicase such as DHX29 [86] or DHX33 [110]. Another common component is the RNA‐binding protein PABP1, which brings the 3′ polyadenosine tail into proximity with the 5′ end [80]. Canonical initiation is therefore greatly enhanced by the presence of a long polyadenosine tail, in contrast to some noncanonical mechanisms.

**FIGURE 3:**
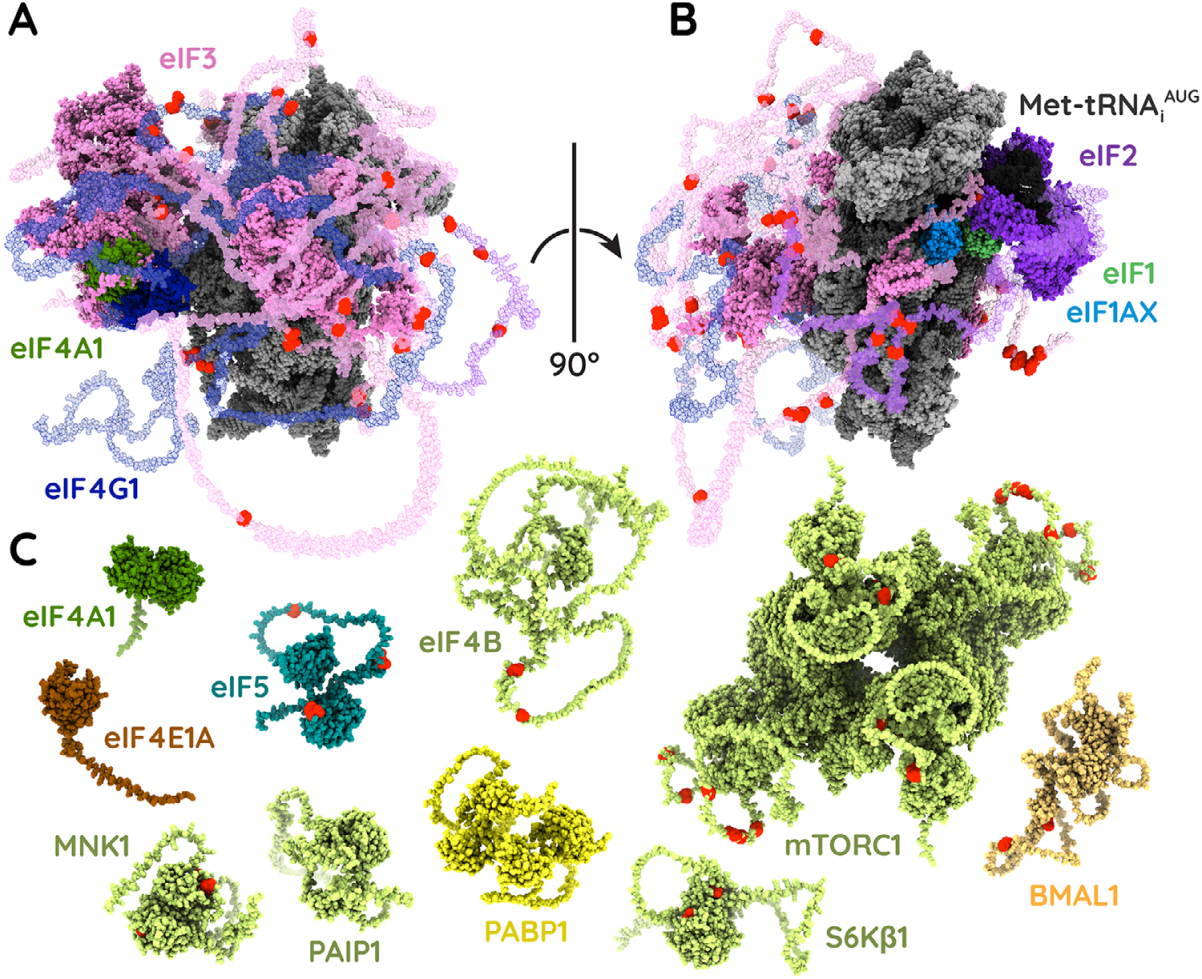
A speculative structural model of the canonical 48S initiation complex, showing views from the (**A**) underside and (**B**) proximal side. This model is based on a recent structure of an intact 48S initiation complex (PDB 6ZMW) [84]. Flexible regions that were not resolvable by electron cryomicroscopy have been modelled using AlphaFold [243, 244] and are shown as translucent. Note that these regions are mobile and mostly predicted to have little or no defined shape; the intention here is to show the complete machinery that must be present in the complex. Almost all the phosphosites (**red**) are in these flexible regions. Panel **C** shows additional proteins which stably associated with the canonical 48S initiation complex. Colors are consistent with Figure 1. All structures were taken from AlphaFold, except mTORC1 which is a hybrid model (PDB 6BCX and AlphaFold) [246]. All proteins are shown to scale with the 48S initiation complex.

The importance of protein phosphorylation for controlling the rate of bulk protein synthesis—and, by extension, the rate of canonical initiation—was first recognized more than three decades ago [111]. A variety of protein kinases mediate this phosphoregulation, but all are in some way responsive to mTOR activity. The best‐characterized downstream effectors of mTOR signalling are the protein kinases S6Kβ1, S6Kβ2, and PASK, which together phosphorylate a wide range of targets including ribosomal protein eS6 [112, 113, 114, 115, 116, 117, 118, 119] and the initiation factors eIF3 [120, 121], eIF4B [120, 122‐125], and possibly eIF4G1 [120, 126, 127]. In the 48S initiation complex, most of these phosphosites are located on the underside of the ribosomal small subunit or in flexible extensions of proteins that attach to the underside (Figure 3) [84]. Though many of these phosphosites are poorly characterized and seem, by themselves, to have little impact on translation [128], mTOR‐directed phosphorylation of multiple residues has been shown to promote canonical initiation by stabilizing contacts between the cap‐binding complex and the 43S preinitiation complex [127, 129] and recruiting additional proteins such as PAIP1 to further enhance translational activity [121].

The close relationship between mTOR and canonical initiation is further highlighted by the direct interactions of mTORC1 and S6Kβ1 with the giant starfish eIF3 [129]. While inactive, S6Kβ1 binds eIF3, occluding mTORC1 and suppressing translation. Upon stimulation of mTOR signalling, however, mTORC1 binds eIF3 [127] and phosphorylates S6Kβ1 in its conserved hydrophobic motif [129]. This causes dissociation of S6Kβ1 from its inhibitory interaction, activation of its kinase domain, phosphorylation of downstream targets, and stabilization of the canonical 48S initiation complex. Based on these data, Holz et al. have speculated that mTORC1 might be a stable component of the 48S initiation complex [129]. Remarkably, just such an interaction has been reported in *Drosophila*, where TORC1 is stably recruited via a combination of eIF4A1·RAPTOR and eIF4G1·Ragulator contacts [130]. When sufficient amino acids are present, the interaction between TORC1 and the 48S initiation complex seems to activate TOR without necessitating any interaction with lysosomes. The interaction also persists when amino acids are depleted, but in this context, eIF4A1 directly recruits the TSC complex to inhibit TORC1. One objection that might be raised against this model is that mammalian cells contain far fewer copies of mTORC1 than initiating ribosomes [131]. However, recent data from murine cells suggest that mTORC1 has at least some specificity for mRNAs such as *c‐myc* and need not interact with ribosomes *en masse* to effectively alter the cellular translatome [132]. Furthermore, though one might expect these supercomplexes to be short‐lived, the interaction between dynein, mTORC1, and the canonical cap‐binding complex is reportedly strong and persistent enough to carry polyribosomes along microtubules for subcellularly localized translation.

A direct link between canonical initiation and circadian rhythms involves the clock protein BMAL1. Despite being well known as a transcriptional activator [9], evidence continues to emerge that, upon phosphorylation at specific residues, BMAL1 and related transcription factors can leave the nucleus to moonlight as cytosolic translation factors. Protein kinases of the PKA·PKG·PKC (AGC) family, including AKT1, S6Kβ1, and their relatives, preferentially phosphorylate serine and threonine residues located within an R*x*R*xx*(S/T)*φ* consensus sequence, where *x* is any residue and *φ* is a bulky hydrophobic residue [133]. BMAL1 and its relatives contain many potential phosphosites with sequence contexts that match or nearly match this consensus, implying that these transcription factors might be extensively phosphorylated in response to mTOR signalling. Indeed, this has been shown for both BMAL1 [5, 134] and CLOCK [135, 136, 137, 138], which are each phosphorylated by multiple AGC kinases. In both cases, phosphorylation disrupts the nuclear localization of the transcription factor, causing its export from the nucleus [5, 134, 138]. Remarkably, upon entering the cytosol, phosphorylated BMAL1 associates with actively translating ribosomes via the canonical cap‐binding complex [5]. In‐vitro translation confirmed that phosphorylated BMAL1—but not naked BMAL1—is a dose‐dependent translational activator, suggesting that BMAL1 might be an authentic component of the canonical 48S initiation complex. The importance of this interaction for circadian translational control remains unclear, however, due to the exceptionally low copy number of BMAL1 compared to initiating ribosomes [131], but it remains possible that BMAL1 might associate preferentially with a defined subset of mRNAs, as is the case for its relative HIF2α [81]. The function of cytosolic CLOCK has received less attention thus far, but it might also interact with ribosomes either by itself or alongside its partner BMAL1.

In addition to activating procanonical factors, whether they be canonical translation factors or moonlighting transcription factors, the protein kinases of the mTOR pathway also inhibit a variety of anticanonical factors which, when freed from mTOR control, selectively repress canonical initiation. Perhaps the best characterized are 4E‐BP1 and its paralogues 4E‐BP2 and 4E‐BP3, which outcompete eIF4G1 for binding to eIF4E1a and prevent assembly of the canonical cap‐binding complex [139]. However, the multitude of alternative cap‐binding proteins, including eIF4E3, eIF3d, PARN, and NCBP2, cannot bind 4E‐BP1 or its relatives, while eIF4E2 binds them only weakly [75], making the 4E‐BP family highly selective inhibitors of canonical initiation. Another protein, PDCD4, interferes directly with the canonical initiation complex by occupying the mRNA channel and inhibiting eIF4A1 at the auxiliary position [140]. Most selective of all are La and the La‐related proteins such as LARP1, which recognize an mRNA motif known as a terminal oligopyrimidine (TOP) and prevent recruitment of the canonical cap‐binding complex to the 5′ UTR [141]. Due to this interaction, the presence of a TOP motif makes an mRNA hyperdependent on mTOR activity for its translation. Thus, in combination with procanonical factors, these anticanonical factors enable mTOR to finely tune the translational landscape of the cell.

Lastly, despite its obvious importance for cellular proteostasis, canonical initiation might not be so canonical after all. In human cells, knockdown of the canonical initiation factor eIF4G1 causes just a ∼20% reduction of bulk protein synthesis, phenocopying mTOR inhibition [142]. The mRNAs that suffer reduced translation under these conditions seemingly represent a distinct class with related functions and similar motifs, yet only represent a small portion of the translatome. On the other hand, knocking down both eIF4G1 and its noncanonical paralogue eIF4G2 reduces bulk protein synthesis by >60%. Despite their major structural and mechanistic differences, these paralogues display a surprising degree of redundancy, with one often able to substitute for the other in what must be a very different initiation complex. The implication is that, though the canonical eIF4G1 controls a significant portion of the translatome, only about a fifth of all proteins rely on canonical initiation exclusively.

We have seen that daily rhythms in bulk translation can be explained by a combination of cell‐intrinsic and ‐extrinsic mechanisms that cooperate to drive rhythmic mTOR activity, peaking in the organismal active phase. However, some proteins are synthesized in the other half of the circadian cycle when mTOR activity is suppressed and the organism is resting and fasting. How is this possible? It is important to remember that mTOR is first and foremost a governor of *translational specificity*. Though mTOR activates the translation of many mRNAs, many others are selectively activated only when mTOR is suppressed [69]. Indeed, mTOR signalling favours the assembly and activation of just one of several possible initiation complexes (Table 1), each of which drives a distinct mechanism of translational initiation. For the rest of this section, we discuss some of the noncanonical mechanisms that take over when mTOR activity is low and explore their possible roles in circadian translational control.

### 3.3 eIF3d‐Mediated Initiation

Though canonical initiation is sometimes assumed to be synonymous with cap‐dependent initiation, many noncanonical mechanisms also require interaction with the 5′ cap. In *eIF3d‐mediated* initiation, for example, the d subunit of eIF3 functionally replaces the canonical cap‐binding protein eIF4E1a [71]. Despite no significant sequence identity, eIF3d is structurally homologous to the cap‐binding ribonuclease DXO and is thought to recognize specific motifs within the 5′ UTR of a bound mRNA.

The noncanonical initiation factor eIF4G2 is also reported to participate in eIF3d‐mediated initiation [143]. This scaffold protein is ubiquitously and abundantly expressed in mammalian tissues [144, 145] and associates with polyribosomes in proliferative cells [146]. It is also implicated in several noncanonical mechanisms of translational initiation and interacts with a wide variety of proteins (Table 1). However, unlike its paralogues in the eIF4G family, eIF4G2 is shorter and cannot bind any members of the eIF4E or PABP families [147], so cap binding and mRNA circularization must be mediated by other proteins or bypassed altogether. Through its interaction with eIF3, therefore, eIF4G2 assembles a noncanonical cap‐binding complex to drive translational initiation.

Several mRNAs that require eIF4G2 for their translation are reported to contain internal‐ ribosome‐entry sites. Though these reports are controversial, to say the least [148, 149], the data nonetheless suggest a role for *cis*‐acting mRNA motifs in noncanonical mechanisms of translational initiation [79, 150]. In the case of eIF3d‐mediated initiation, the *cis*‐acting motif is an eIF3d‐binding stem‐loop within the 5′ UTR [151, 152, 153]. Several mRNAs are predicted to contain this stem‐loop and have been shown to crosslink with eIF3d, including *JUN*, *CDK12*, *RAPTOR*, *PER1*, and *NR1D1*. In the case of *JUN*—the best‐characterized example—disruption of the stem‐loop reduces both eIF3d binding and translational activity.

The hypothesis that the clock proteins PER1 and NR1D1 are synthesized via an eIF3d‐dependent mechanism has not yet been tested, but there is evidence for some kind of noncanonical translation of these mRNAs. For example, translation of *PER1* is upregulated by LARK1 and LARK2 (also known as RBM4A and RBM4B, respectively), which bind the 3′ UTR and presumably mediate circularization of the *PER1* mRNA in place of PABP1 [154, 155]. The name *lark* comes from the *Drosophila* orthologue of these proteins, which disrupts circadian phenotypes when mutated [156]. These proteins reportedly display rhythmic abundance at the protein level in mice [155] and *Drosophila* [157], but not at the mRNA level [155, 158], suggesting that circadian control of LARK1 and LARK2 might contribute to rhythmic noncanonical initiation. Notably, LARK1 has also been implicated in hypoxic initiation, where it joins other noncanonical initiation factors to circularize the mRNA and stimulate cap‐dependent translation during hypoxia [81].

Though these studies suggest a possible link between circadian rhythms and noncanonical initiation, the most striking evidence comes from the casein kinase CK2, which phosphorylates eIF3d, disrupts its interaction with the 5′ cap, and suppresses eIF3d‐mediated initiation [152]. A heteromeric protein kinase, CK2 is a widely expressed and highly conserved regulator of many cellular processes, including both the cell cycle [159] and the circadian cycle [160]. A peculiar property shared by all three mammalian paralogues of the catalytic subunit—α1, α2, and α3—is that intramolecular constraints hold the kinase domain in an active conformation in all solved structures regardless of context. As a result, the catalytic subunits are constitutively active, meaning that their *catalytic activities* are impervious to stimuli such as post‐translational modifications, second messengers, and regulatory proteins [159, 161]. Nevertheless, the *catalytic specificities* of CK2 variants are rhythmic in vivo [162], due in part to regulatory proteins such as RNA helicases [163], growth factors [164, 165], and the regulatory subunit CK2β [162, 166‐167].

**FIGURE 4:**
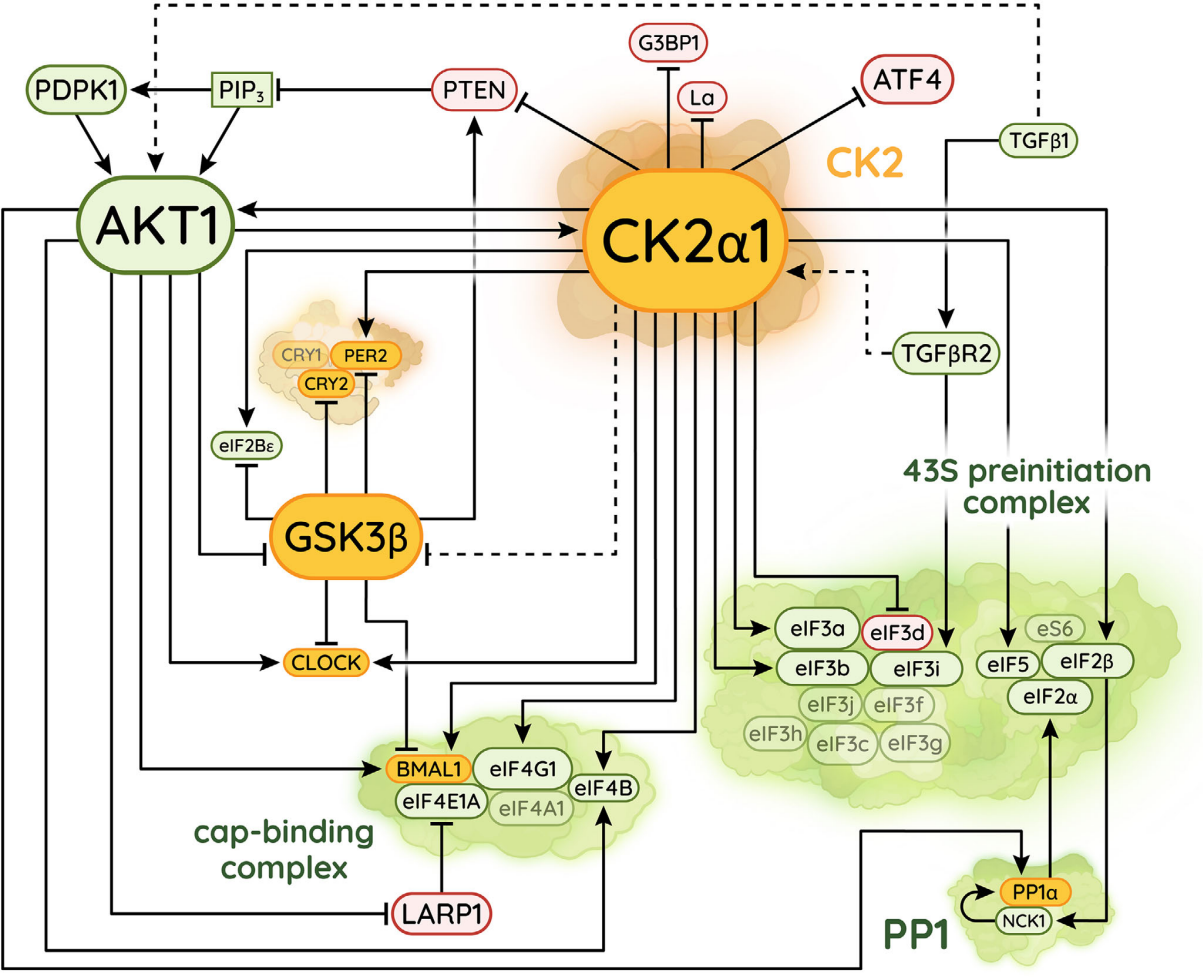
The CK2 signalling pathway, highlighting interactions that regulate canonical initiation. The colour scheme is the same as in Figure 2. Though they are heavily interconnected, we have separated the CK2 and mTOR pathways into separate figures for simplicity.

There is substantial overlap between the CK2 and mTOR signalling pathways (Figure 4). Like mTOR activity, the growth‐promoting activities of CK2 are stimulated by serum [168], glucose [169], insulin [170, 171], and other growth factors [164‐165, 172]. Under such conditions, CK2 phosphorylates the tumour suppressor PTEN, thereby relieving inhibition of the protein kinase PDPK1 and indirectly activating the master kinases AKT1 and mTOR [173, 174, 175, 176]. Acting more directly to promote growth, CK2 also binds [177] and phosphorylates [178] AKT1, increasing the duration of AKT1 activity [179, 180, 181]. AKT1 in turn phosphorylates CK2, enhancing the activity of CK2 towards specific substrates [182]. As well as mutually activating each other, CK2 and mTOR share many of the same targets, with CK2 reportedly phosphorylating more than three hundred proteins [183] including the stress‐granule nucleator G3BP1 [184], the stress‐response factor ATF4 [185], the clock proteins BMAL1 [162, 167], CLOCK [186], and PER2 [187, 188], the anticanonical factor La [189], and the translational initiation factors eIF2β [190], eIF2Bε [191], eIF4B, eIF4G1 [192], eIF5 [193], eIF3a, eIF3b [192], and, of course, eIF3d [152]. The combined effect of these phosphorylations is to suppress stress‐response pathways and stimulate the mTOR‐directed canonical mechanism of translational initiation. Indeed, much like those of mTOR, the phosphosites targeted by CK2 have been shown to activate translation by stabilizing contacts between canonical initiation factors in plants [194].

We hypothesize, therefore, that growth‐promoting CK2 activities occur with a circadian rhythm in phase with rhythmic mTOR activity. As such, it is noteworthy that CK2 phosphorylates eIF3d in nutrient‐rich cell culture while mTOR is also active [152]. By contrast, during acute glucose starvation [152] or targeted mTOR inhibition [195]—both of which are expected to suppress growth‐promoting CK2 activities—eIF3d escapes phosphorylation and drives noncanonical translation. This pattern implies that eIF3d‐mediated initiation might be rhythmic under constant conditions and out of phase with respect to mTOR and CK2.

### 3.4 Leaky Scanning and Reinitiation

The textbook paradigm that eukaryotic genes are monocistronic, containing only a single open reading frame (ORF) in contrast to the polycistronic operons of bacteria and archaea, is not quite accurate. Truly polycistronic genes have been identified throughout the Eukarya, including in mammals [196], while about half of all mammalian genes contain upstream ORFs (uORFs) [197]. A typical uORF is short, having a median length of 48 to 78 nt, with a start codon in the 5′ UTR and a stop codon either in the 5′ UTR or inside and out of frame with respect to the downstream main ORF (mORF) [198]. Though some uORFs encode functional oligopeptides, uORFs primarily function as *cis*‐acting translational regulators of their corresponding mORFs. In particular, the presence of a uORF in an mRNA necessitates noncanonical translation of the downstream mORF in the form of two mechanisms: *leaky scanning* and *reinitiation*. Though these mechanisms are distinct, with different requirements for *trans*‐acting proteins and *cis*‐acting mRNA motifs, we group them under the same heading because both can occur on the same mRNA and their relative activities seem to be regulated circadianly.

According to the classic model of translational initiation, the 48S initiation complex assembles at the 5′ cap before scanning along the mRNA in the 5′‐to‐3′ direction [68]. As it moves, the complex probes the codon–anticodon interaction and the surrounding nucleobases until it finds the appropriate start codon, usually an AUG triplet within a region approximating a Kozak consensus sequence. Upon finding the start codon, eIF2γ hydrolyses GTP and the ribosome commits to translation of the corresponding ORF. However, despite the attractive simplicity of this model, start‐codon recognition is not always so clear cut. Not all legitimate start codons match the Kozak consensus, while many 5′ UTRs contain multiple AUG, CUG, GUG, or other near‐cognate triplets with favourable sequence contexts [199]. This variability leads to the phenomenon of *leaky scanning*, whereby different ribosomes commit to translational elongation from different start codons on the same mRNA. If the alternative start codons are in frame with each other without an intervening stop codon, multiple isoforms of the encoded protein are synthesized with different N‐termini. If the mRNA contains a uORF, however, leaky scanning can permit translation of the downstream mORF instead of the uORF. *Reinitiation*, by contrast, allows translation of both the uORF and the mORF. Uniquely among initiation mechanisms, reinitiation can be thought of as a modification of the recycling phase of translation. After termination at a stop codon, the ribosomal large subunit and the tRNA on the penultimate codon must be removed, as occurs in canonical recycling, but in reinitiation the mRNA remains associated with the ribosomal small subunit, a new Met‐tRNA_i_^AUG^ is recruited, and scanning resumes downstream of the uORF.

Though the proportion of initiation events that occur at a particular start codon is largely determined by sequence context, the cell is also able to regulate such events via *trans*‐acting factors. These include the usual suspect eIF4G2, which displaces eIF4G1 and eIF4E1a from the scanning complex to facilitate leaky scanning [200] and has similarly been implicated in reinitiation [201]. Both mechanisms therefore seem to involve capless cap‐binding complexes centred around eIF4G2. This noncanonical scaffold has no discernible effect on start‐codon readthrough but seems to be required for efficient recognition of downstream start codons, especially when the scanning complex meets obstacles such as stem‐loops or a queue of elongating ribosomes in a uORF that might impede scanning and favour dissociation of eIF4G1 [202, 203]. The same obstacles might also explain why RNA helicases such as DHX9, DHX36, and DDX3X are often required for leaky scanning. The canonical initiation factor and RNA helicase eIF4A1 is poorly processive and easily stalled by secondary structures in the path of the scanning complex [204]. Many ribosomes therefore recruit potent auxiliary helicases to push past these secondary structures and initiate at downstream start codons. When these auxiliary helicases are depleted from cells, mORF translation is suppressed while uORF translation markedly increases, with this effect being specific to structured mRNAs [205, 206, 207]. Several RNA helicases, most notably DDX3X, have also been implicated as regulators of circadian rhythms [208] and of the casein kinases CK1ε and CK2 [163, 209], suggesting a possible mechanistic link to the circadian clock.

In contrast to other initiation mechanisms, which tend to be regulated at the recruitment step, both leaky scanning and reinitiation are primarily regulated at the point of start‐codon recognition. Ordinarily, in the absence of codon–anticodon pairing, the Met‐tRNA_i_^AUG^ is held in place by the eIF2 heterotrimer, which binds the ribosomal small subunit via its α and β subunits. The evolution of this complex seems to have been a prerequisite for the scanning mechanism that is unique to eukaryotes. As such, eIF2 participates in most eukaryotic mechanisms of translational initiation, including leaky scanning and reinitiation, but it is not the only protein that can fulfil this function. The canonical initiation factor eIF5B, which usually binds the mature 48S initiation complex to recruit the ribosomal large subunit, can also recruit Met‐tRNA_i_^AUG^ during hypoxic initiation [210], leaderless initiation [211], and internal ribosome entry [212] in a similar manner to its bacterial orthologue IF2. Of more general utility, however, are the three partially redundant translation factors eIF2A, eIF2D, and MCT1·DENR. Each of these can recruit Met‐tRNA_i_^AUG^ to the ribosomal small subunit [213, 214, 215], but eIF2A [216] and eIF2D [214] also recruit Leu‐tRNA^CUG^ and Val‐tRNA^GUG^, respectively. CUG, GUG, and UUG are the most common non‐AUG start codons found in uORFs and are recognized poorly by eIF2, so eIF2A and eIF2D are thought to be important regulators of many uORF‐containing mRNAs [199, 217].

In common with many noncanonical initiation factors, eIF2A, eIF2D, and MCT1·DENR tend to be used when mTOR activity is suppressed [198‐199, 217], implying an antiphasic relationship with rhythmic mTOR signalling. Most interesting from a circadian perspective is the MCT1·DENR heterodimer—ribosome profiling has confirmed the presence of actively translated uORFs in several rhythmically translated mRNAs, with *CLOCK* mRNA, in particular, being dependent on DENR for translation of its mORF [218]. DENR seems to be a dedicated reinitiation factor, with depletion of DENR having little effect on uORF translation but severely reducing downstream mORF translation [219, 220].

The interplay between eIF2 and its noncanonical rivals is best illustrated by *ATF4* mRNA, which contains two uORFs that together control translation of the downstream mORF. Under stress‐free nutrient‐rich conditions, translation of uORF1 is quickly followed by reinitiation at uORF2 [221]. Because uORF2 overlaps in a different frame with the mORF, the translation of uORF2 blocks mORF translation. However, in response to cellular stresses such as amino‐acid starvation or viral infection, the pool of active eIF2 is depleted and the incidence of leaky scanning increases, leading to reinitiation further downstream at the mORF and synthesis of ATF4. Notably, translation of the *ATF4* mORF seems to involve both leaky scanning and reinitiation—rather than just leaky scanning through both uORF1 and uORF2—because it requires either eIF2D or MCT1·DENR [222, 223].

The ATF4 protein itself is a transcription factor and the architect of a signalling pathway known as the integrated stress response (ISR), directing a programme of gene expression that allows the cell to survive periods of stress [221]. In mammals, four protein kinases are responsible for activating the ISR, though only one of them—GCN2—is conserved among other eukaryotes. Each of these kinases phosphorylates S52 on the α subunit of eIF2, simultaneously blocking its interaction with the ribosomal small subunit and strengthening its interaction with the eIF2B complex. Because eIF2B acts as a guanosine exchange factor for eIF2, replacing GDP with GTP in the active site of the γ subunit, phosphorylation of the α subunit turns eIF2 into a competitive inhibitor of its own activity [224]. The subsequent depletion of GTP‐bound eIF2 reduces bulk translation and, at the same time, alters the properties of leaky scanning and reinitiation to activate translation of specific mRNAs such as *ATF4*.

Not all ISR signalling occurs in response to stress. Remarkably, eIF2α phosphorylation follows a circadian rhythm, which has been reported in *Neurospora* [225, 226] and mammals [227] and is likely conserved among other eukaryotes. The mechanistic basis of circadian eIF2α phosphorylation is still poorly characterized in mammals but is somewhat better understood in the multicellular fungus and circadian model organism *Neurospora*. The activity of Cpc3 (the *Neurospora* orthologue of GCN2) is circadian, peaking during the day, with this rhythm being necessary for rhythmic eIF2α phosphorylation [225]. There is no concomitant rhythm in Cpc3 abundance; instead, recent data suggest that rhythmic accumulation of uncharged tRNA is at least partially responsible for the rhythmic activation of Cpc3 [228]. This is especially interesting because the classic model of GCN2 activation, first characterized in the distantly related *Saccharomyces*, depends on acute amino‐acid starvation, but a circadian rhythm of aminoacylation goes some way to explaining how eIF2α might be phosphorylated under constant nutrient‐rich conditions.

There is currently no direct evidence for rhythmic GCN2 activity in mammals or other species, though it is worth noting that deletion of *GCN2* from mice severely damps the circadian rhythm of eIF2α phosphorylation [227]. Relevant observations can also be drawn from *Arabidopsis*, in which the abundances of amino acids and other metabolites oscillate under constant light [229], while GCN2 activity is sensitive to light, amino acids, and osmolarity [230]. Given the extreme conservation of GCN2 among eukaryotes, it is plausible that rhythmic GCN2 activity might be a common feature of circadian translational control.

Alongside GCN2, circadian ISR signalling would also require clock‐controlled dephosphorylation of eIF2α. In mammals, the protein phosphatase PP1 dephosphorylates eIF2α at S52 and is therefore the primary antagonist of the ISR [221]. The catalytic subunit of PP1 assembles into a heteromeric holophosphatase with a regulatory subunit such as PP1‐R15A or PP1‐R15B [231]. The R15A subunit is expressed in response to eIF2α phosphorylation in stressed cells and mediates the recovery phase of the ISR by forming a negative‐feedback loop that downregulates the ISR after a delay. The R15B subunit, on the other hand, is constitutively expressed and maintains low levels of eIF2α phosphorylation under constant conditions. Both subunits contain an eIF2α‐binding motif that mediates the recruitment of PP1 to its target. Though PP1‐R15A is dispensable, deletion of PP1‐R15B from mice or the single PP1‐R15 from *Drosophila* results in embryonic lethality [232, 233]. The importance of PP1 activity for maintaining low levels of eIF2α phosphorylation is also shown by *Saccharomyces*, which, though it lacks an orthologue of PP1‐R15, has an N‐terminal extension on eIF2γ that directly recruits PP1 to the eIF2 heterotrimer [234]. The same N‐terminal extension also exists in *Neurospora* and mediates circadian recruitment of PP1, such that dephosphorylation of eIF2α peaks at night [226]. Whether a similar rhythm occurs in mammals remains to be seen, though the PP1‐R15A negative‐feedback loop suggests a possible mechanism.

## 4 A Hypothesis for Circadian Translational Control

Throughout our exploration of circadian translational control, we have described four mechanisms of translational initiation governed by three highly conserved master kinases: mTOR, CK2, and GCN2. Both mTOR and CK2 are activated in response to nutrients and growth factors, meaning that their activities oscillate with a peak during the organismal active phase when feeding happens [6, 167]. The activity of GCN2, though less well characterized, is rhythmic in *Neurospora* [225] and is activated by fasting typical of the organismal rest phase [221]. Though reinforced by systemic cues, the rhythms of mTOR, CK2, and GCN2 persist under constant conditions.

Each master kinase controls its own intracellular signalling cascade, but the three cascades share so many components that they can be thought of as a continuous signalling pathway. The term *pathway*, by analogy to roadmaps in day‐to‐day life, is used to describe a sequence of interconnected causal steps which traces the flow of something—in this case *information*—through a biological system [235]. Importantly, a pathway itself says nothing about the direction or timing of flow but predicts outcomes based on different inputs. From this perspective, mTOR signalling, CK2 signalling, and the ISR are contiguous branches of a single homeostatic pathway. It is through this pathway that the circadian clock controls the various mechanisms of translation and maintains cellular proteostasis.

**FIGURE 5:**
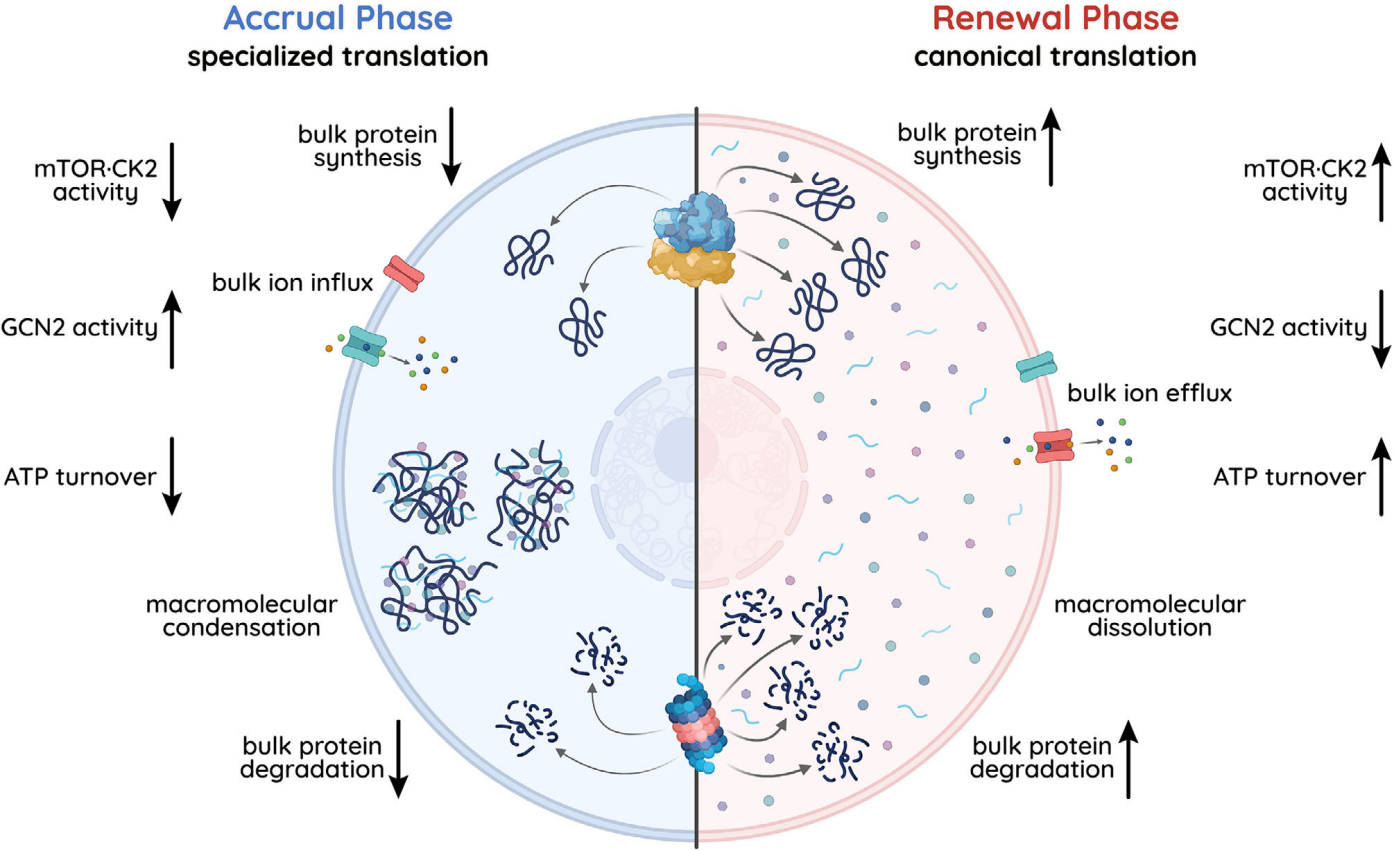
A two‐phase model for cell‐autonomous circadian proteostasis. In the **accrual phase**, GCN2 suppresses bulk protein synthesis but activates noncanonical mechanisms of translational initiation. This phase of specialized translation modestly alters the composition of the proteome, potentially allowing the cell to survive periods of anticipated nutrient deprivation during the organismal rest phase. In the **renewal phase**, mTOR and CK2 increase the rate of bulk protein synthesis by selectively activating canonical initiation. At the same time, the rate of bulk protein degradation is increased to maintain the total protein content at a roughly constant level, leading to rapid protein turnover and proteomic renewal.

Flux through the mTOR·CK2·GCN2 pathway never reaches steady state; instead, homeostasis is maintained through oscillations in the relative activities of the master kinases. This means that information flows through the pathway in different directions at different times. The antagonistic relationship between mTOR·CK2 on the one hand and GCN2 on the other leads us to propose a biphasic model of circadian proteostasis (Figure 5):

In the *accrual phase*, GCN2 and other factors suppress bulk protein synthesis while selectively upregulating noncanonical mechanisms of translational initiation. This phase of *specialized translation* modestly alters the composition of the proteome, allowing the cell to survive as it accrues insults during a period of reduced nutrient availability, typically coinciding with the rest phase for peripheral tissues (daytime for mice, nighttime for humans). Bulk protein degradation continues at a slow rate, occurring mostly via autophagy due to suppression of mTOR activity. The slow rate of protein synthesis and suppression of mTOR signalling also lead to reduced macromolecular crowding and increased cytoplasmic fluidity, as many proteins and RNAs are sequestered into condensates and membranous compartments [6, 236‐237]. Cell volume and osmotic pressure are maintained by crowding‐associated compensatory transport of ions (CACTI), which increases the ion content of the cytosol to counteract decreased crowding [6, 238]. Stress granules and other condensates contribute to the suppression of bulk protein synthesis but might also facilitate specialized translation and spatiotemporally localized assembly of protein complexes [239].

In the *renewal phase*, an increase in mTOR·CK2 activity selectively upregulates canonical initiation and accelerates the rate of bulk protein synthesis. For peripheral tissues, this begins around the transition to increased locomotor activity and feeding. At the same time, autophagy is suppressed while the rate of bulk protein degradation accelerates to maintain the total protein content of the cell at a roughly constant level [7]. This combination of faster synthesis and faster degradation leads to rapid protein turnover and proteomic renewal, facilitated by increased macromolecular crowding as proteins and RNAs are liberated from condensates and membranous compartments. Other processes such as bulk transcription are also upregulated, while CACTI maintains osmostasis through net ion efflux.

The primary function of these two phases—and perhaps the primary function of circadian rhythms in general—is the temporal compartmentalization of protein synthesis. Being the most expensive process that happens in most cells, the circadian clock prioritizes the efficiency of protein synthesis, with all other processes ultimately being subservient to the function of efficiently maintaining proteostasis. Much like the YROs of *Saccharomyces*, which are similarly driven by rhythmic flux through the mTOR·CK2·GCN2 pathway but out of synchrony with the solar cycle [55], circadian rhythms might have evolved to compress the synthesis and degradation of most proteins into a defined window of time. This window coincides with an increase in anticipated nutrient availability due to daily feeding rhythms, thereby optimizing the efficiency of every step required for protein synthesis, from glucose catabolism and tRNA aminoacylation to complex assembly and post‐translational modification.

Under typical experimental conditions, the activities of the master kinases never fall to zero. In *Neurospora*, for example, circadian eIF2α phosphorylation—taken as a marker for Cpc3 activity—peaks at about 30% relative to acute eIF2α phosphorylation induced by amino‐acid starvation [225]. This is a significant proportion with major consequences for proteostasis but still represents a minority of eIF2α proteins and a milder phenotype than that induced by acute stress. Therefore, TOR and CK2 must remain at least partially active throughout the circadian cycle in *Neurospora*. Similarly, bioluminescent reporters in mammalian cell cultures show that cycling clock proteins such as PER1 and PER2 never cease to be synthesized even when their rates of synthesis fall to their lowest values [240]. The amplitudes of circadian oscillations are therefore small compared to what is observed in response to acute stresses but still large enough to alter cellular and organismal phenotype and to be conserved throughout eukaryotic evolution.

Several predictions from this hypothesis need to be tested. For example, the timing of the accrual and renewal phases is expected to vary between the brain and peripheral tissues, and their relative durations should vary with environmental conditions. Moreover, it remains to be seen whether the transition from one phase to the other is gradual or sudden; a sudden change with stochastic phase variation between cells would appear gradual at the population level and could only be resolved by longitudinal live‐cell microscopy. On a more mechanistic level, further work is needed to characterize the various cap‐binding complexes that control translation and to confirm whether noncanonical mechanisms such as eIF3d‐mediated initiation are indeed under clock control. And most mysterious of all are the cell‐autonomous oscillations that drive rhythmic mTOR·CK2·GCN2 flux and the synchronization of protein synthesis with protein degradation.

## 5 Concluding Remarks

We have proposed that the primary function of circadian rhythms is the temporal compartmentalization of protein synthesis. By compressing the synthesis and degradation of most proteins into a brief window of time, the circadian clock generates bursts of proteomic renewal that optimize the energy efficiency of every step in the lifecycle of a protein. Old proteins are cleared at the same time as their replacements are synthesized, ensuring that amino acids are available for protein synthesis and suppressing proteotoxic fluctuations in protein abundance. This temporal compartmentalization is especially pertinent for the assembly of heteromeric macromolecular complexes, amounting to about half of the proteome [60], which must be strictly controlled in space and time to avoid metabolic inefficiency and proteotoxic stress associated with supernumerary subunit production.

We have also speculated on the mechanisms underlying this temporal compartmentalization. Circadian control of protein synthesis seems to be achieved by rhythmic flux through signalling pathways centred around the protein kinases mTOR, CK2, and GCN2. These kinases post‐translationally modify the translational machinery, activating alternative initiation mechanisms such that different mechanisms take precedence at different times. Each mechanism involves recognition of specific mRNA motifs, so the choice of mechanism determines which mRNAs are translated at any given time.

Building on these ideas, we have proposed a biphasic model of circadian proteostasis comprising alternate phases of accrual and renewal. The accrual phase is characterized by reduced metabolic activity and increased rates of noncanonical translation. By contrast, the renewal phase is dominated by canonical translation and rapid proteomic turnover.

Our proposals have major implications for the scientific understanding of cellular homeostasis and its perturbation in ageing and disease. As is well known, disruption of circadian rhythms is strongly associated with a range of debilitating conditions such as neurodegeneration, cancer, obesity, diabetes, cardiovascular disease, and depression [241]. Notably, these same diseases are also associated with aging, which is thought to involve a gradual loss of circadian control. It seems plausible that dysregulation of circadian proteostasis might be a critical common factor underlying the aetiology of these diseases. Therefore, a mechanistic understanding of circadian translational control will be necessary to develop better therapeutic strategies and to help people live healthily for longer.

## Conflicts of Interest

The authors declare no conflicts of interest.

## Data Availability

Data sharing is not applicable to this article as no new data were created or analyzed in this study.
